# Meta-analysis of studies using metformin as a reducer for liver cancer risk in diabetic patients

**DOI:** 10.1097/MD.0000000000006888

**Published:** 2017-05-12

**Authors:** Shujuan Ma, Yixiang Zheng, Yanni Xiao, Pengcheng Zhou, Hongzhuan Tan

**Affiliations:** a Department of Epidemiology and Health Statistics, Xiangya School of Public Health, Central South University; b Viral Hepatitis Key Laboratory of Hunan Province, Department of Infectious Disease, Xiangya Hospital, Central South University, Changsha, China.

**Keywords:** diabetes mellitus, liver cancer, meta-analysis, metformin

## Abstract

Metformin has garnered more interest as a chemo-preventive agent given the increased liver cancer risk in diabetic patients. This work was undertaken to better understand the effect of metformin use on liver cancer risk in diabetic patients.

A comprehensive literature search was performed in PubMed, Embase, BIOSIS Previews, Web of Science, and Cochrane Library through July 30, 2016. Meta-analyses were performed using Stata version 12.0, with odds ratio (ORs) and 95% confidence intervals (CIs) as effect measures.

Twenty-three studies were included. Meta-analysis of 19 studies involving 550,882 diabetic subjects suggested that metformin use reduced the ratio of liver cancer by 48% (OR = 0.52; 95% CI, 0.40–0.68) compared with nonusers. The protective effect was validated in all the exploratory subgroup analyses, except that pooled result of post hoc analyses of 2 randomized controlled trials found no significant difference between subjects with metformin and those without, with OR being 0.84 (95% CI, 0.10–6.83). After adjusting for hepatitis B/C virus infection, cirrhosis, obesity, behavioral factors, and time-related bias, the association was stable, pooled OR ranged from 0.42 to 0.75.

A protective effect for liver cancer was found in diabetic metformin users. However, more randomized clinical evidence is still needed to verify the results.

## 1 Introduction

Liver cancer is the fifth most common cancer worldwide and the third-leading cause of cancer related-death.^[1]^ Diabetes mellitus (DM) is becoming an established independent risk factor for liver cancer as reported in multiple observational studies and subsequent meta-analyses.^[2,3]^ In these existing studies, DM has been reported to confer a 2- to 4-fold risk of liver cancer, and the risk increases with DM severity and duration. However, this risk may be mitigated by antidiabetic medications (ADMs). Metformin, a widely used ADM, has recently attracted great attention for antitumor effect in a wide range of malignancies including liver cancer, through both insulin-dependent and insulin-independent mechanisms.^[4]^ However, the evidence for a cancer preventive effect for metformin has not been consistently demonstrated.

Association of metformin and risk of liver cancer are mainly studied in animal and observational human studies. A meta-analysis demonstrated that metformin appeared to have a direct antihepatocellular carcinoma (HCC) effect in animal models.^[5]^ Accumulating epidemiologic studies comparing the risk of liver cancer between those using metformin with those using other ADMs have shown somewhat variable results,^[6–9]^ and it was also endorsed that confounders were not well addressed in most studies.^[2]^ Several meta-analyses have been published to determine if a consistent effect of metformin use on liver cancer incidence was evident.^[10–13]^ Except for the incomplete included studies, meta-analyses in previous reviews were rough, and the heterogeneity was not explored in detail.

In our opinion, the differences in estimates and the heterogeneity between studies could largely be explained by differences in study designs, quality, population, the comparators used, estimation of the exposure to metformin (duration and dosage) and adjusted factors, as the inability to account for these factors may result in certain degrees of bias. To better understand the association of metformin and risk of liver cancer, we embarked on a systematic review and meta-analysis with integrated overall, subgroup and sensitivity analyses.

## 2 Methods

### 2.1 Inclusion criteria

Either observational studies (cohort and case–control studies) or post hoc analyses of randomized controlled trials (RCTs) were included if they evaluated and defined exposure to metformin or biguanide, reported liver cancer incidence or related outcomes of diabetic patients, provided effective comparison groups, and reported hazard ratio (HR)/relative risk (RR)/odds ratio (OR) and corresponding 95% confidence intervals (CIs), or provided sufficient data for their estimations. Inclusion was not restricted by language, study size, or publication type. The most recent or most comprehensive report was given precedence if there were multiple publications (regardless of study design) from the same population, while the others might be included in subgroup analysis according to the concrete conditions.

### 2.2 Literature search

A comprehensive literature search was performed in PubMed, Embase, BIOSIS Previews, Web of Science, Cochrane Library, National Institutes of Health database, EU clinical trials register from the earliest date available through July 30, 2016, without any restrictions. In order to include more potential literature, our overall search strategy only included terms for metformin and liver cancer. The comprehensive literature search was conducted as follow: ((liver cancer) OR (liver carcinoma) OR (liver neoplasm) OR (liver tumor) OR (hepatoma) OR (hepatocellular carcinoma) OR (HCC) OR (hepatic cancer) OR (hepatic neoplasm) OR (hepatic tumor) OR (cholangiocarcinoma)) AND ((metformin) OR biguanide). We screened bibliographies of selected original studies, review articles, and relevant conference abstracts. Attempts were made to contact the corresponding authors for additional data.

### 2.3 Data extraction

Citations were merged together in Endnote, version X7 to facilitate management. Two authors independently applied the inclusion criteria to all retrieved articles in an unblinded standardized manner, evaluated by title, abstract, and full text. For each of eligible study, information of first author, publication year, location, study design, data source, study period, mean follow-up, characteristics of study population (mean age, sex ratio), definition of exposure and control, dose and duration of exposure (if reported), comparison groups, risk estimates (included HR, RR, OR), and 95% CIs with and without adjustment for confounding factors were selectively extracted onto piloted structured forms independently by 2 authors. As subjects in most studies used combination therapy, the final analysis on exposure used the dichotomous categorical variable of “with” or “without” use of metformin.

Adjusted factors were extracted, and some of them were selected for further analysis: infected with hepatitis B virus (HBV) or hepatitis C virus (HCV), cirrhosis, obesity (including body mass index and obesity), behavioral factors (including alcohol abuse and cigarette smoking), use of statins, and time-related bias (including DM duration, duration of exposure, duration of follow up, enrollment date, time of first ADM prescription, calendar time, and other time-dependent factors). Any disagreements during study selection or data collection were resolved by discussion, referring back to the original article.

### 2.4 Quality assessment

Newcastle–Ottawa Scale ^[14]^ was used to assess the quality of observational studies and post hoc analyses of RCTs, which were treated as cohort studies to achieve quality assessment. In this scale, studies were scored across 3 categories: selection of subjects (4 stars), comparability of study groups (2 stars), and assessment of outcome/exposure (3 stars). Star rating system was used to indicate the quality, with a maximum of 9 stars: 0 to 5 stars as low quality and 6 to 9 stars as high quality.

### 2.5 Statistical analysis

Adjusted estimate was mainly used for quantitative analysis. Crude estimate served as an alternative in case of no adjusted estimate was available. When estimates or 95% CIs were missing or incomplete, appropriate summary statistics or Kaplan–Meier curves were used to calculate based on published methods.^[15]^ OR was employed as a common measure of the association between metformin use and liver cancer risk due to the enrollment of case–control studies in most analyses. Between-study heterogeneity was qualitatively assessed by using Cochrane Q test with a significance level of *P* ≤ .1, and quantified by estimated I^2^ (I^2^ < 50% representing low heterogeneity, 50% ≤ I^2^ ≤ 75% representing moderate heterogeneity, I^2^ > 75% representing substantial heterogeneity).^[16]^ An inverse variance fixed-effects model was used to calculate when the test for heterogeneity was not statistically significant, otherwise the DerSimonian–Laird random-effects model was employed.^[17]^

Sensitivity analyses were performed to assess the robustness of results. Between-study sources of heterogeneity were further investigated using subgroup analyses by stratifying original estimates according to study characteristics (study design, setting, and quality), controlled ADM, and adjustment. Analyses of adjusted estimates were emphasized on studies controlling for HBV/HCV infection, cirrhosis, obesity, behavioral factors, use of statins, and time-related bias, given their modifying effects on metformin's activity on DM and liver cancer risk.^[18–20]^ Publication bias was detected for overall analysis using Begg test and Egger test (publication bias considered present if *P* ≤ .1).^[21,22]^ All the statistical analyses were 2-sided and performed using Stata version 12.0 (StataCorp, College Station, TX).

## 3 Results

### 3.1 Description of included studies

**Figure 1:**
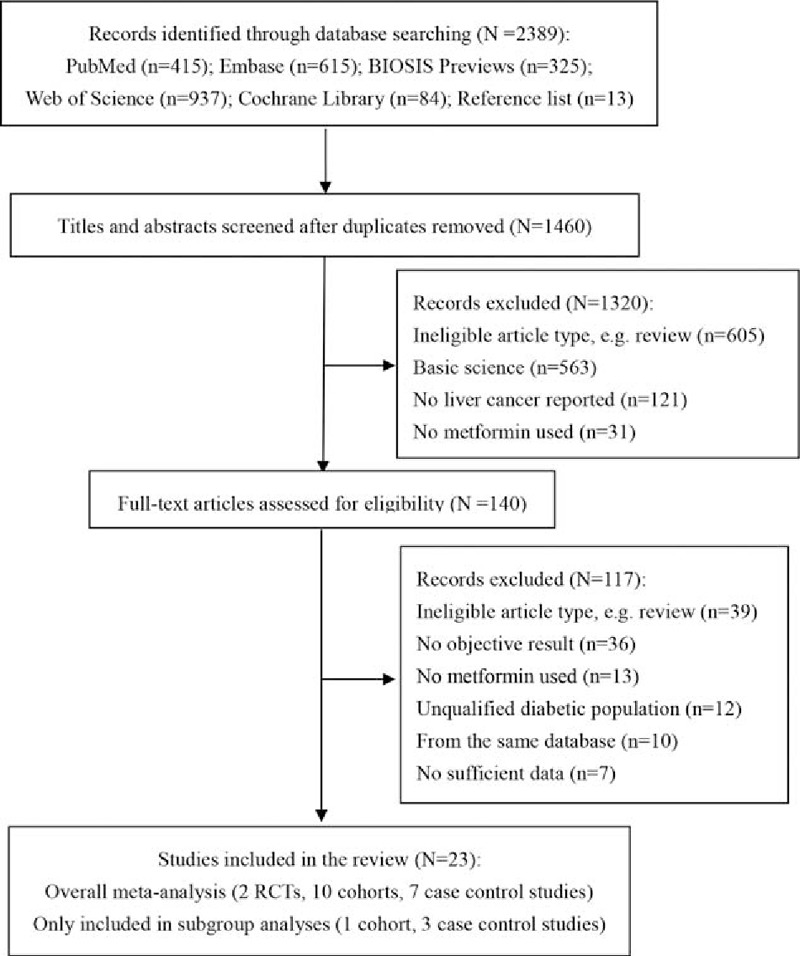
PRISMA flow diagram of study selection. RCT = randomized controlled trials.

Searches identified 2389 potentially relevant studies. The selection process is shown in Fig. 1. Twenty-three studies fulfilled the inclusion criteria and were included in the meta-analysis (2 RCTs, 11 cohort studies, 10 case–control studies). These 23 studies cumulatively reported more than 35,000 cases of liver cancer in 663,335 diabetic subjects. Only 19 studies were included in the overall analysis,^[6–9,23–36]^ the remaining 4 studies^[37–40]^ were only included in subgroup analyses for specific conditions, as they were multiple publications from the same populations. In fact, more multiple publications were found during the study selection. Eleven studies^[7,27,37–39,41–46]^ (6 cohort and 5 case–control) were conducted in Taiwan, China using the National Health Insurance data, and hence only 2^[7,27]^ of them with different time period (ignoring a coinciding year) were included in our overall analysis, and 3^[37–39]^ were just included in different subgroup analyses. Likewise, 3 Italian case–control studies^[32,47,48]^ were from a same cohort, and only one^[32]^ of them was included. Moreover, data of 1 cohort^[31]^ and 1 case–control study^[40]^ were both from the United Kingdom Clinical Practice Research Datalink, only the cohort with larger sample size and higher quality was included in our overall analysis.

The characteristics of included studies are shown in Table 1 . Twelve studies^[8,23,25,26,29,30,32–36]^ were population-based studies, and the remainder^[6,7,9,24,27,28,31]^ were hospital-based studies. Overall methodological quality of included studies was high. Treatment comparators were sulfonylureas,^[28,31,32,37]^ insulin^[25,26,29,32,37,40]^ or nonuse of any ADMs.^[25,26,40]^ Type of liver cancer was clearly informed to be HCC in most studies. In addition to age and sex, most studies adjusted for HBV/HCV infection,^[7,24,25,27,30,32,33,40]^ obesity,^[7,25,31,32]^ behavioral factors,^[8,24,25,31–33]^ use of statins,^[6,31,38]^ and time-related bias.^[6,7,9,25,28,31,32]^

**Table 1:**
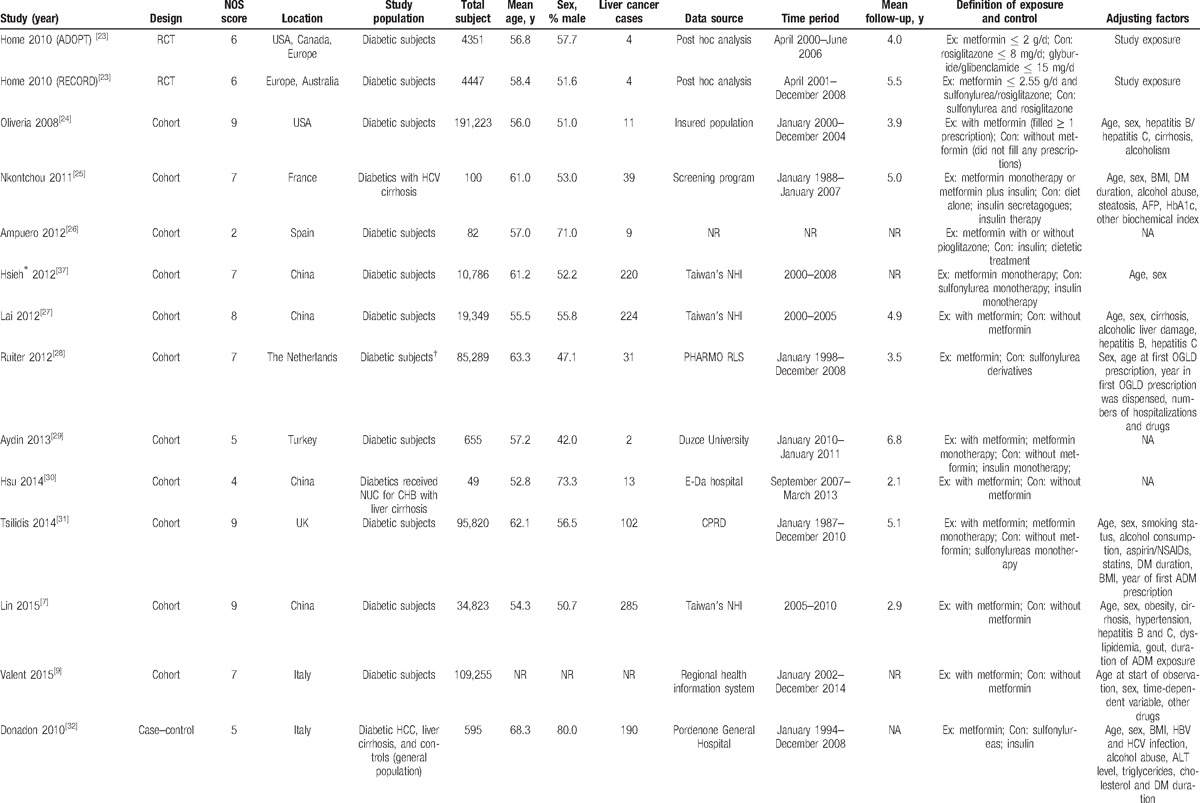
Characteristics of included studies in the meta-analysis.

**Table 1 (Continued):**
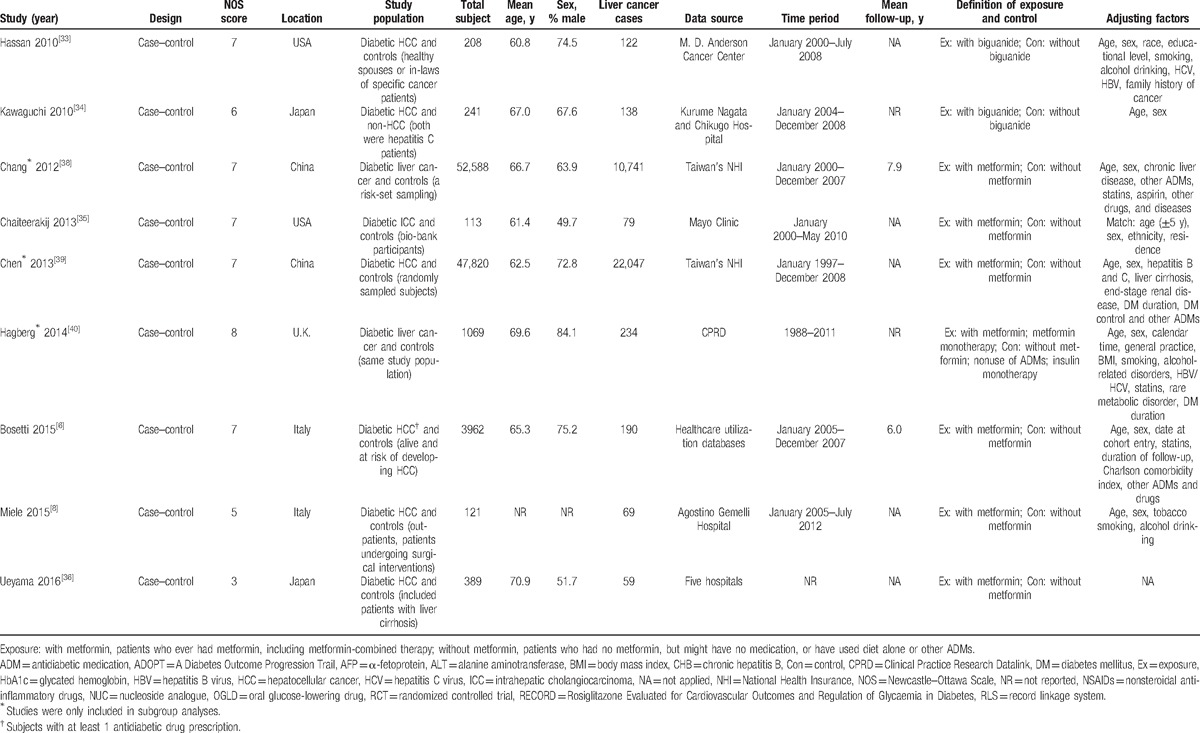
Characteristics of included studies in the meta-analysis.

### 3.2 Overall analysis

**Figure 2:**
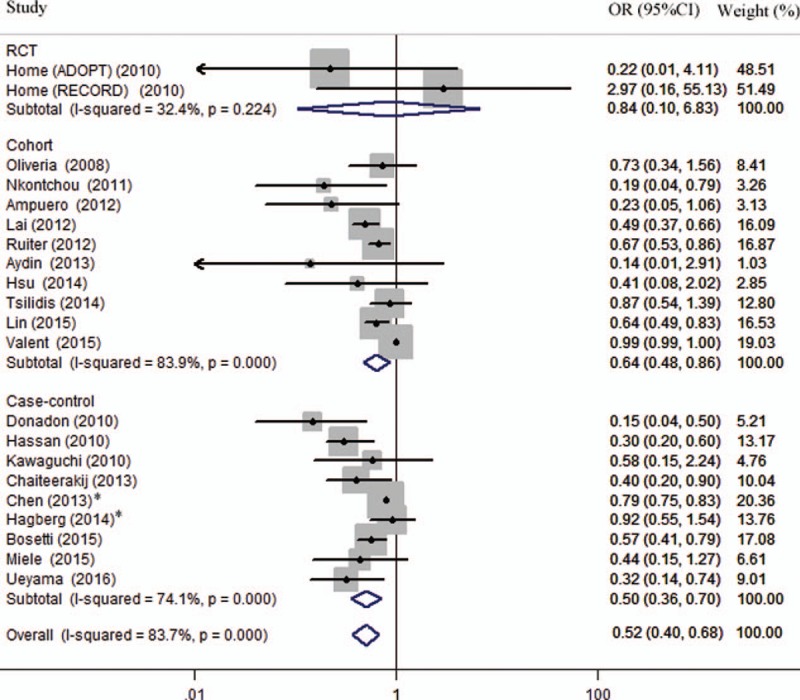
Forest plot of the association between use of metformin and liver cancer risk in diabetic patients. RCT = randomized controlled trials. ∗Studies were multiple publications and they were only included in corresponding subgroup analysis.

On the basis of 19 studies^[6–9,23–36]^ involving 550,882 diabetic patients, compared with metformin nonusers, metformin use reduced the ratio of liver cancer by 48% (OR = 0.52; 95% CI, 0.40–0.68; *P* < .001), with substantial heterogeneity (I^2^ = 83.7%) (Fig. 2). Sensitivity analysis using leave-one-out method found that the pooled result was robust when omitting any one study alone, heterogeneity kept substantial except when omitting the study^[9]^ with maximum weight from overall analysis, I^2^ dropped to 33.5%, with the summary OR being 0.53 (95% CI, 0.44–0.63; *P* < .001). Significant publication bias was found for overall analysis by Begg test (*P* = .069) and Egger test (*P* < .001).

### 3.3 Subgroup analysis

Subgroup analyses were conducted to further validate the result from overall analysis, and to explore potential sources of heterogeneity among studies (Table 2). Hierarchies of study setting, quality, controlled drugs, and adjustment did not change over the significant reduction in ratio of liver cancer in metformin users. Pooled result of post hoc analyses of 2 RCTs^[23]^ found no significant difference between subjects with metformin and those without, with OR being 0.84 (95% CI, 0.10–6.83; *P* = .871) (Fig. 2). Subgroup analyses of hospital-based studies with relatively small sample size (OR = 0.32; 95% CI, 0.24–0.44) or studies with low quality (OR = 0.29; 95% CI, 0.18–0.49) showed an exaggeration in metformin's effect. Metformin showed higher protective effect of liver cancer when compared with insulin (OR = 0.36; 95% CI, 0.25–0.51), other than sulfonylurea (OR = 0.65; 95% CI, 0.55–0.78) and nonuser of any ADM (OR = 0.62; 95% CI, 0.40–0.98).

**Table 2:**
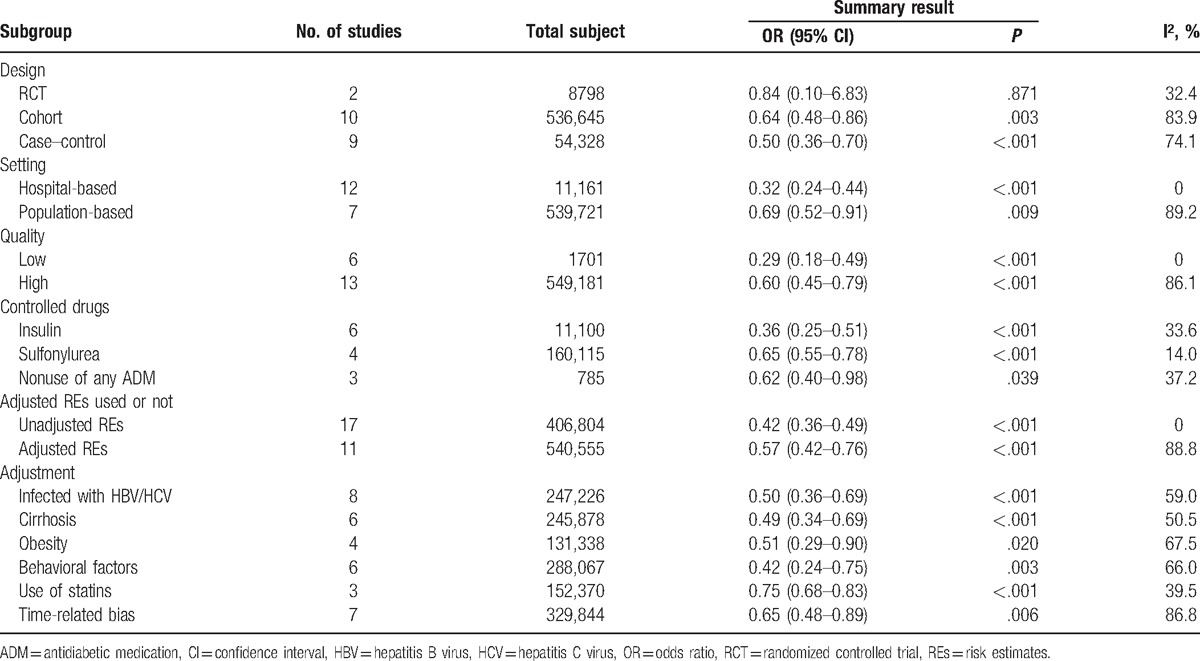
Summary results of subgroup analyses.

Furthermore, use of adjusted estimates caused numerical increases on pooled OR and heterogeneity. Compared to the analysis of all adjusted estimates (OR = 0.57; 95% CI, 0.42–0.76; *P* < .001), numerical increases in the ratio of liver cancer in metformin users were found when the estimates were adjusted for HBV/HCV infection, cirrhosis, obesity, and behavioral factors (pooled ORs ranged from 0.42 to 0.51), while the ratio reduction decreased in studies adjusted for use of statins (OR = 0.75; 95% CI, 0.68–0.83; *P* < .001) and time-related bias (OR = 0.65; 95% CI, 0.48–0.89; *P* = .006).

Heterogeneity was significant in most subgroups, with I^2^ (>50%) ranging from 50.5% to 89.2% (Table 2). Nevertheless, no heterogeneity was found in the subgroup analyses of hospital-based studies (I^2^ = 0%) and studies with low quality (I^2^ = 0%). Moreover, for subgroup analyses of RCTs, studies adjusted for use of statins, and when the controlled drugs were definitely restricted to insulin, sulfonylurea, or nonuse of any ADM, heterogeneity was limited (I^2^ ranged from 14.0% to 39.5%).

## 4 Discussion

This systematic review synthesized evidence on association between use of metformin and risk of liver cancer in diabetic patients from 23 studies. We used systematic strategy and broad search terms in multiple databases to identify as many studies as possible. Rigorous methods were used to extract and appraise the data. Multiple publications from the same population were checked in any analysis. Considering the potential confounding factors for liver cancer, adjusted estimates were used instead of the unadjusted ones as much as possible to make the summary results more precise and plausible.

Overall meta-analysis of 19 studies involving 550,882 diabetic subjects found that, relative to nonuse, use of metformin reduced the ratio of liver cancer by 48% (OR = 0.52; 95% CI, 0.40–0.68; *P* < .001), with substantial heterogeneity (I^2^ = 83.7%). Sensitivity analysis found that the heterogeneity was mainly from a high-quality population-based study,^[9]^ with maximum weight, but without reporting the exact number of liver cancer cases. After omitting this study, heterogeneity of overall analysis was significantly decreased (I^2^ dropped from 83.7% to 33.5%), so was the heterogeneity in subgroup analyses of high-quality studies (I^2^ dropped from 86.1% to 33.7%) and population-based studies (I^2^ dropped from 89.2% to 7.3%). The beneficial effect of metformin was validated in observational studies, with a diminution in cohort studies (OR = 0.64) and a rise in case–control studies (OR = 0.50), but it lost significance in RCTs (OR = 0.84; 95% CI, 0.10–6.83; *P* = .871), which might be largely limited by the fewer available studies (n = 2). Actually, most RCTs are not designed or sufficiently powered to examine cancer outcomes due to the short follow-up periods and very few cancer events.^[31]^

Metformin is one of the most commonly prescribed medications in the treatment of DM. However, DM treatment is a dynamic process, ADMs might be changed continuously and often used in combination,^[29]^ which made definition of exposure using dichotomous categorical variable of “with” or “without” use of metformin be somewhat less convincing. Thus we further subanalyzed supplemented comparisons between monotherapy of ADMs. Results showed that metformin had higher protective effect of liver cancer when compared with insulin, other than sulfonylurea and nonuser of any ADM. Beyond the plausible finding that use of insulin increased risk of liver cancer,^[44]^ another explanation is that metformin is a first-line ADM prescribed in less severe or shorter duration of DM, while insulin is usually prescribed to patients with longer duration and more advanced DM, which in turn may be associated with higher risk of liver cancer.^[49]^ However, when compared to nonuser of any ADM (mild or newly diagnosed DM patients), monotherapy use of metformin achieved a 38% (OR = 0.62; 95% CI, 0.40–0.98) reduction in ratio of liver cancer, probably reflecting the real world scenario.

Lots of confounders may have modifying effect on association between metformin and liver cancer risk in diabetic patients. Presence of DM in patients with cirrhosis is an independent factor for the progression to liver cancer.^[50]^ Moreover, metformin may be specifically sensitive to certain etiological types of liver cancer.^[10]^ After adjusting for HBV/HCV infection, cirrhosis, obesity, and behavioral factors, the beneficial effects on the ratio of liver cancer for metformin use were significant and larger (pooled OR ranged from 0.42 to 0.51), which might be the true link between metformin use and liver cancer risk in diabetic patients. Recent reviews underscored the prevalence of time-related bias in observational studies, potentially leading to inflated estimates of metformin's protective effect.^[19]^ Time-related bias includes immortal-time bias, time-window bias, and time-lag bias.^[19]^ Of note, exclusion of time-biased studies from our analysis resulted in a numerical decline on the ratio reduction (OR = 0.65; 95% CI, 0.48–0.89; *P* = .006). Thus further studies should take these biases into account in the study design and analysis.

Statins was previously found to be associated with a reduced risk of liver cancer.^[51]^ Most of included studies did not take the concomitant use of statins into account to adjust for potential confounding. Subgroup analysis of studies adjusted for the use of statins caused a numerical decline on the ratio reduction (OR = 0.75; 95% CI, 0.68–0.83; *P* < .001), which might suggest a synergistic effect of metformin and statins for liver cancer, in addition to their dose-dependent protective effects.^[46,52]^ Given the rising disease burden of liver cancer, looking for chemo-preventive strategy is necessary, especially for cheap nonetiology-specific medications, like metformin and statins, still with favorable safety profile.^[52]^ However, further researches are needed to establish definitive role of metformin and statins on the prevention of liver cancer in diabetic patients.

The observational nature allows only an association to be established. Plenty of experimental studies have added evidence to metformin's protective effect on malignancies. Although the exact mechanism is not fully understood, several biologically plausible mechanisms have shown that metformin might have direct antiliver cancer activity by inhibiting proliferation and colony formation ability through adenosine monophosphate-activated protein kinase (AMPK) in HCC cells^[53]^; suppressing HCC cell growth through induction of cell cycle G1/G0 phase arrest, p21CIP and p27KIP expression, and down-regulation of cyclin D1^[54]^; inducing apoptosis in HCC cells via signaling pathways, including AMPK and p38 mitogen-activated protein kinase^[55]^; and suppressing xenograft tumor growth in mouse models.^[56]^ Moreover, as an antihyperglycemic agent and insulin sensitizer, metformin treatment inhibits hepatic gluconeogenesis,^[57]^ reduces serum concentrations of insulin and insulin growth factor I,^[58]^ improves glycemic control, and decreases inflammatory response,^[59]^ thus leading to less aggressive behavior of cancer cells. However, given that not all in vitro and in vivo work with animal models could be successfully translated into clinical outcomes in humans, well-designed RCTs are still needed to provide authentic evidence.

Several limitations of this study needed to be addressed and merited further discussion. First, significant heterogeneity was presented between studies in some of our analyses. However, sensitivity analyses found that the heterogeneity could be mostly interpreted by 1 same article.^[9]^ Except for the contribution of heterogeneity, omitting this article would not change over the initial results. Second, information on treatment was obtained through prescriptions contained in patients’ medical records, therefore a gap between prescribed and actual dose could bias the results. Third, adjustments of included studies might be incomplete and inconsistent. Although we performed subgroup analyses of adjusted estimates controlled for several important factors. Some other confounders were failed to control, such as information like details of DM (severity and duration) and metformin use (dose and duration) were absent in most studies, which would have been important to adjust for residual confounding.^[9]^ Fourth, significant publication bias was found for overall analysis. However, this might probably be the small-study effect rather than true publication bias, especially in the presence of significant heterogeneity among studies.^[60]^

## 5 Conclusion

In conclusion, a protective effect in the risk of liver cancer was found in diabetic metformin users, and the protective effect was validated in most of our exploratory analyses. However, the conclusion should be interpreted with caution given the possibility of residual confounding. Simultaneously, limited by the observational study design, a conclusion of causality cannot be drawn. Clinical trials are needed to determine if the observations in diabetic subjects can be expanded to a wider range of population, and then to reveal the true scenario in real life.
